# Local arterial stiffness measured by ultrafast ultrasound imaging in childhood cancer survivors treated with anthracyclines

**DOI:** 10.3389/fcvm.2023.1150214

**Published:** 2023-06-06

**Authors:** Rahna Rasouli, Jerome Baranger, Cameron Slorach, Wei Hui, Maelys Venet, Minh B. Nguyen, Matthew Henry, Josh Gopaul, Paul C. Nathan, Luc Mertens, Olivier Villemain

**Affiliations:** ^1^ Division of Cardiology, Department of Pediatrics, The Hospital for Sick Children, University of Toronto, Toronto, ON, Canada; ^2^ Division of Hematology/Oncology, Department of Pediatrics, The Hospital for Sick Children, University of Toronto, Toronto, ON, Canada

**Keywords:** anthracycline, arterial stiffness, ultrafast ultrasound imaging, cancer survivor, pediatrics

## Abstract

**Background:**

There is conflicting literature regarding the long-term effect of anthracycline treatment on arterial stiffness. This study assessed local arterial stiffness using ultrafast ultrasound imaging (UUI) in anthracycline treated childhood cancer survivors, at rest and during exercise.

**Methods:**

20 childhood cancer survivors (mean age 21.02 ± 9.45 years) treated with anthracyclines (mean cumulative dose 200.7 ± 126.80 mg/m^2^) and 21 healthy controls (mean age 26.00 ± 8.91 years) were included. Participants completed a demographic survey, fasting bloodwork for cardiovascular biomarkers, and performed a submaximal exercise test on a semi-supine bicycle. Pulse wave velocity (PWV) was measured in the left common carotid artery by direct pulse wave imaging using UUI at rest and submaximal exercise. Both PWV at the systolic foot (PWV-SF) and dicrotic notch (PWV-DN) were measured. Central (carotid-femoral) PWV was obtained by applanation tonometry. Carotid measurements were taken by conventional ultrasound. Measures were compared using two-tailed Students *t*-test or Chi-squared test, as appropriate.

**Results:**

There was no statistically significant difference (*p* > 0.05) between childhood cancer survivors and healthy controls in demographic parameters (age, sex, weight, height, BMI), blood biomarkers (total cholesterol, triglycerides, LDL-c, HDL-c, hs-CRP, fasting glucose, insulin, Hb A1c), cardiovascular parameters (intima media thickness, systolic and diastolic blood pressure, heart rate, carotid diameters, distensibility) or PWV measured by UUI at rest or at exercise. There was also no difference in the cardiovascular adaptation between rest and exercise in the two groups (*p* > 0.05). Multivariate analysis revealed age (*p* = 0.024) and LDL-c (*p* = 0.019) to be significant correlates of PWV-SF in childhood cancer survivors, in line with previously published data.

**Conclusion:**

We did not identify a significant impact of anthracycline treatment in young survivors of childhood cancer on local arterial stiffness in the left common carotid artery as measured by UUI.

## Introduction

Every year, approximately 1,000 Canadian children are diagnosed with cancer (1). More than 50% of these patients are treated with anthracyclines, which are known cardiotoxic agents (2, 3). While the exact mechanism is unclear, cardiotoxicity is thought to be related to oxidative stress and mechanisms resulting in apoptosis (4). The short- and long-term cardiovascular effects of anthracycline therapy have been a topic of research for many years. Multiple studies have demonstrated increased long-term cardiac risk in childhood cancer survivors (CCS), particularly with higher risks for developing left ventricular dysfunction and congestive heart failure (5–8). Over the last decade, emerging studies have reported vascular changes in young childhood cancer survivors, suggesting early vascular changes may predispose to development of early atherosclerosis (9). The increased incidence of atherosclerosis has been hypothesized to be related to possible direct vascular effects of anthracycline treatment or indirect effects related to inflammation resulting from the cancer and its treatment (10). However, the literature is conflicting, and there is no consensus on the impact and extent of vascular damage.

**Figure 1:**
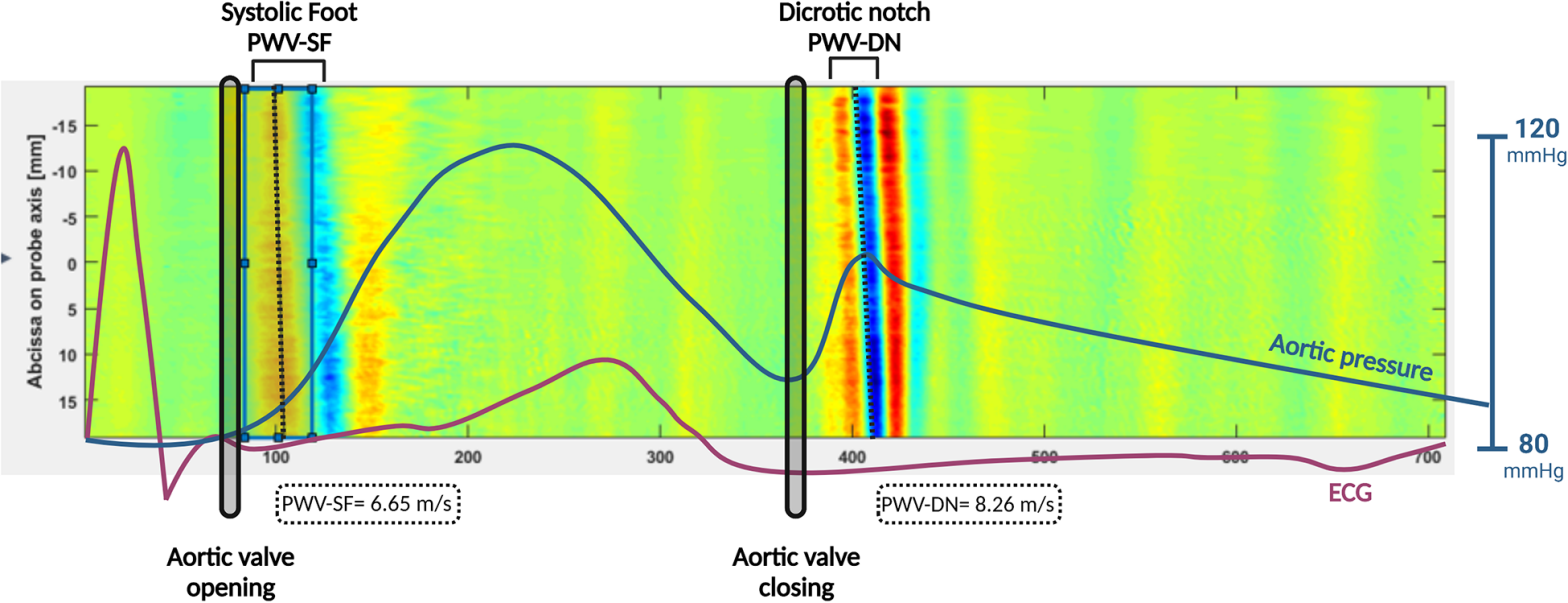
PWV-SF and PWV-DN measurement using UUI. 2D space-time graph representing displacement of superficial common carotid artery wall. *X*-axis represents time in the form of frame number. *Y*-axis represents position of arterial wall segment in relation to the ultrasound probe. Red represents movement towards probe. Blue represents movement away from the probe. Sloped vertical lines represent pulse waves. Dotted lines represent the slope of pulse wave, thus representing the velocity. Purple line represents ECG tracing, and blue line represents aortic pressure, time matched to space-time graph. Aortic valve opening and closure are represented by black vertical ellipses.

Arterial stiffness is a biomarker of vascular health and a predictor of cardiovascular events in adults (11). There are different methods that can be used to evaluate arterial stiffness, most often based on tonometry or B-mode based measurements of stiffness, which have significant limitations (12). In the clinical setting, pulse wave velocity (PWV) is used as a non-invasive surrogate for arterial stiffness. Ultrafast ultrasound imaging (UUI) is a newer method to measure local arterial PWVs with high accuracy (13, 14). The very high temporal resolution allows for the detection of small amplitude displacements with direct visualization of the pulse wave propagating along the vessel walls (15). This provides a direct measurement of local PWV which reflects local arterial stiffness in a specific arterial segment. UUI allows for the visualisation of distinct forward-propagating pulse waves at different times in the cardiac cycle: PWV at the systolic foot (PWV-SF), after aortic valve opening, and PWV at the dicrotic notch (PWV-DN), after aortic valve closure (see Figure 1). These parameters estimate different stiffness states of the arterial wall during the cardiac cycle.

The aim of the current study was to use UUI to measure local PWV in the left carotid artery at rest and during an exercise in childhood cancer survivors treated with anthracyclines and compare the findings to healthy controls. The hypothesis was that local arterial stiffness in CCS would be increased at rest and demonstrate an abnormal response to exercise. This is the first study evaluating arterial stiffness in childhood cancer survivors using UUI.

## Methods

### Subjects

This study included 20 childhood cancer survivors (CCS) and 21 healthy volunteers (HV). Participants were recruited by the department of echocardiography at the Hospital for Sick Children, Toronto. The study was approved by the research ethics committee (REB #1000060834) and all participants gave informed consent. The CCS group inclusion criteria were: age <18 years at time of cancer diagnosis, currently in remission from cancer, and treated with anthracyclines with the final dose completed at least 3 years before inclusion. No participants had significant congenital heart disease or cardiomyopathy. Healthy volunteers were age-matched and had no history of cardiovascular disease.

### Study protocol

**Figure 2:**
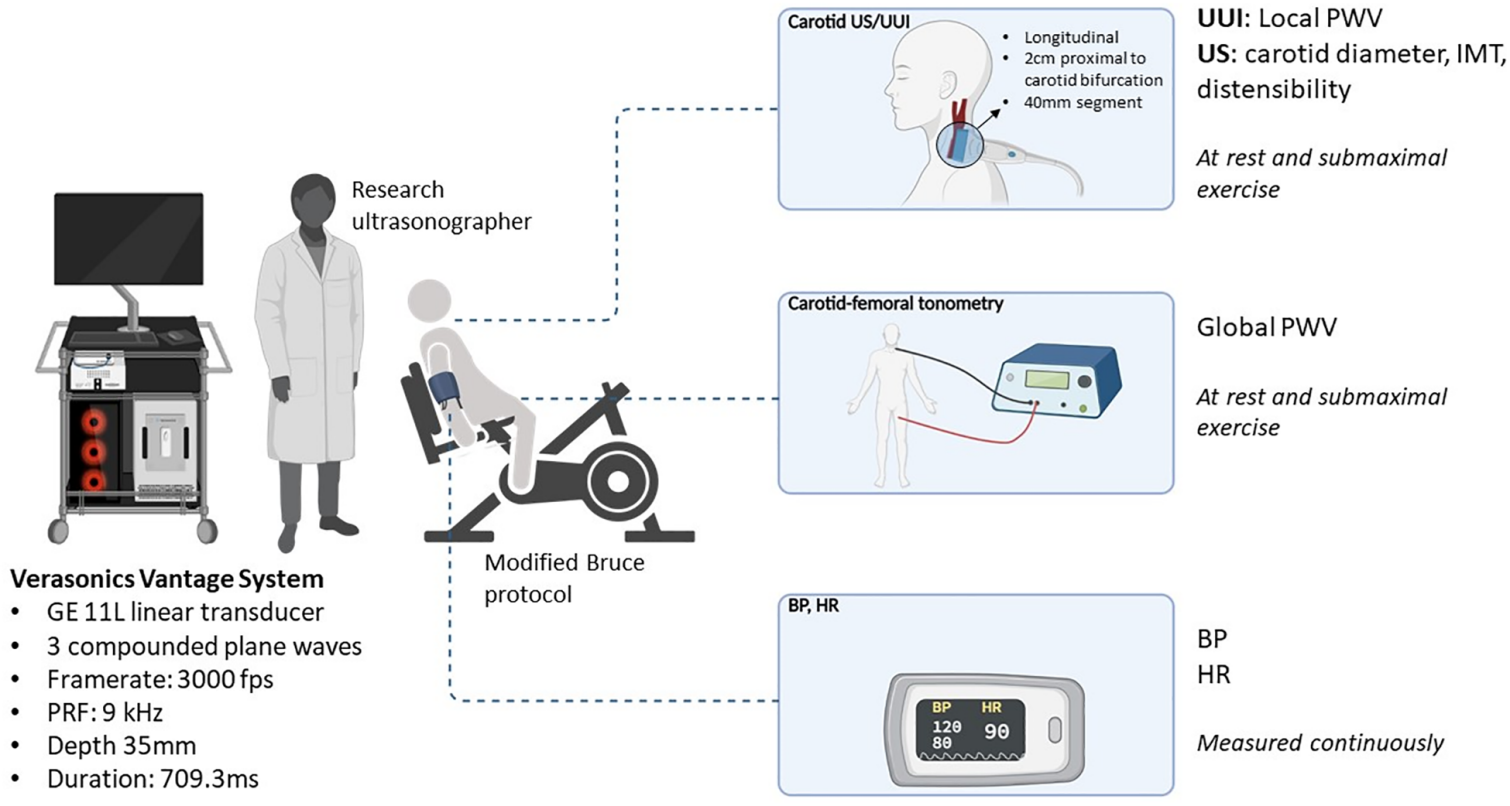
Study protocol. Diagram representation of study protocol. Participants underwent an exercise trial on a semi-supine bicycle ergometer such that patients were sitting and leaning back at about a 120° angle with their neck stable and exposed. Their arms were free and resting on arm rests such that blood pressure measurements could be consistently taken. BP measurements on the right arm were taken at each increase in exercise load following the Bruce protocol. There was a continuous 12-lead electrocardiogram (ECG) including heart rate monitoring. PWV measurements (by UUI and tonometry), as well as carotid parameter measurements were taken at rest and submaximal exercise.

Demographic parameters including age, sex, weight, and height were obtained. Blood pressure (systolic: SBP; diastolic: DBP) measurements, conventional ultrasound acquisitions of the left common carotid artery (CCA), UUI acquisitions of the left CCA, and carotid-femoral PWV measurements by applanation tonometry on the right were taken at baseline (rest, seated position) and at submaximal exertion during an exercise test, at 75 W (see Figure 2). Offline analysis of carotid artery imaging was done to obtain carotid intima-media thickness (IMT), carotid diameter (at SBP/peak systole and DBP/end-diastole), and distensibility, both at rest and submaximal exercise.

### Bloodwork

Participants who consented in advance to blood work fasted overnight. Measurements included total cholesterol, triglycerides, low density lipoprotein cholesterol (LDL-c), high density lipoprotein cholesterol (HDL-c), fasting glucose, HbA1c, insulin, and high sensitivity C-Reactive protein (hs-CRP).

### Blood pressure assessment

A Dinamap® ProCare oscillometric device (GE Healthcare, Tampa, Florida, USA) was used to measure systolic, diastolic, and mean arterial pressure and simultaneous heart rate in triplicate. Established guidelines for BP measurement were followed with measurements in the right arm using an appropriate cuff size with the arm at heart level after 5 min of rest with the participant seated (16). Oscillometric readings above the 90th percentile for gender, age and height were repeated with an auscultatory technique (17).

### Exercise test

A modified Bruce Protocol was used on a semi-supine bicycle ergometer (Ergoline USA), such that patients were sitting and leaning back at about a 120° angle with their neck stable and exposed (depicted in Figure 2) (18). Their arms were free and resting on arm rests such that blood pressure measurements could be consistently taken. BP measurements on the right arm were taken at each increase in exercise load following the Bruce protocol. There was a continuous 12-lead electrocardiogram (ECG) including heart rate monitoring.

### Conventional ultrasound

Carotid artery imaging was performed using a GE Vivid-E95 Ultrasound system (GE Healthcare, USA) equipped with a 11l linear transducer. Carotid IMT and carotid artery distensibility were assessed (see Figure 2). The acquisition was performed on left CCA. A 2D cine-loop of the common carotid artery including proximal bulb was recorded. An M-mode 1 cm proximal to the bulb was generated. This was assessed by the same skilled ultrasonographer (19). Offline analysis was performed using automated analysis software (Carotid Analyzer, Medical Imaging Applications LLC, USA). Carotid diameter was measured at peak systole (maximum diameter) and at end-diastole (at peak R wave on ECG). Arterial borders were decided by lumen-wall interface. Zooming and increasing sweep speed were used to improve measuring accuracy. Three measurements were recorded and averaged.

### Carotid-to-femoral PWV measurement (tonometry)

Central (aortic) PWV was obtained by applanation tonometry (SphygmoCor, AtCor Medical Pty Ltd, West Ryde, Australia). It was measured as the transit time of the pulse wave between the right carotid and right femoral artery (20). The transit time was measured automatically by the software as the difference between the onsets of the waveforms between simultaneously interrogated sites. The distance between sites were measured to the closest millimeter with a tape measure. PWV was derived using the formula: PWV = transit distance/transit time (m/s). The mean of two measurements was used in all analyses.

### PWV assessed by UUI

The left CCA was assessed by UUI using a programmable ultrafast ultrasound system (Vantage 256, Verasonics, Kirkland, WA, USA) equipped with a GE 11l linear transducer (GE Vingmed, Horten, Norway). The probe was placed in a longitudinal orientation with its distal end about 2 cm proximal to the carotid bifurcation of the CCA. The length of the arterial segment was about 40 mm. After the probe was set up and the sequence was initiated, the UUI acquisition was triggered automatically by the peak of the R wave on ECG. Each acquisition lasted 709 ms and covered at least one cardiac cycle. Each measurement was taken at least twice to ensure reproducibility. Raw ultrasound data were beamformed into In-phase/Quadrature datasets (IQ), and processed using MATLAB R2019a (The MathWorks Inc., Natick, MA, USA). Offline analysis of PWV on MATLAB used well verified post-processing methods, as follows. The arterial walls were first localized on the IQ images. The anterior and posterior CCA were manually delineated and approximated by a second order polynomial line. The radial velocity of the wall was then derived by a frame-to-frame IQ auto-correlation (Loupas estimator) (15, 21). The velocity of the anterior arterial wall segment over time could then be displayed ass 2D space-time graph, on which the different pulse-waves appear as tilted colored stripes (Figure 1). The slope of these color stripes corresponded to the PWV and was obtained using a local Radon transforms of the space-time graph as in (22). Within a cardiac cycle, two pulse waves can be seen: one from the beginning of systole created by the pressure wave of blood being pumped out (systolic foot, PWV-SF), and one at the end of systole formed by the pressure differential from the aortic valve closing (dicrotic notch, PWV-DN).

### Statistical analysis

Results are presented as mean ± standard deviation (SD). Normality was defined by the Shapiro-Wilk test. Parametric measures were compared using unpaired two-tailed or paired Students *t*-test, or Chi-squared test, as appropriate. Non-parametric measures were compared with the Mann–Whitney *U* test. Correlations were analyzed using Pearson correlation analysis. Multiple regression was performed on independent variables showing a linear correlation to PWV to identify significant determinants of PWV. All statistical analyses were performed using MedCalc (MedCalc Software Ltd, Ostend, Belgium). A *p*-value <0.05 was considered to indicate statistical significance. Bonferroni correction was applied where suitable.

## Results

### Population characteristics and clinical parameters

This study included 20 CCS treated with anthracyclines, aged 20.9 ± 9.2 years, and 21 HV, aged 26 ± 8.91 years (*p* = 0.10). Table 1 outlines the study population characteristics, including demographics, clinical parameters in survivors, cancer diagnosis, and blood biomarkers. In the CCS population, the mean age at diagnosis was 7.0 ± 3.6 years, the mean duration since treatment completion was 13.7 ± 7.6 years, and the mean total anthracycline dose (in doxorubicin equivalents) was 200.7 ± 126.80 mg/m^2^. Only six CCS participants (30%) had an anthracycline dose exceeding 250 mg/m^2^. None of the participants had significant known cardiac abnormalities or familial cardiomyopathies, pre-existing cardiovascular conditions, kidney disease, or type 1 diabetes. They were all non-smokers. Five participants (25%) received radiation therapy. No participants were receiving or had previously received cardiac medications, other than one participant who received Dexrazoxane. Table 2 summarizes cardiovascular parameters at rest and exercise, respectively. No significant differences were found between the groups in any of the measured parameters, including blood biomarkers.

**Table 1:** Population characteristics.

Parameter	Cancer survivor (*n* = 20)	Control (*n* = 21)	*p*
**Demographic parameters**			
Age (years)	21.02 ± 9.45	26.00 ± 8.91	0.10
Sex (M/F)	12M/8F	12M/9F	0.85
Weight (kg)	67.8 ± 15.3	70.15 ± 17.97	0.67
Height (cm)	166.7 ± 9.35	170.63 ± 7.48	0.16
BMI (kg/m^2^)	24.3 ± 4.6	24.00 ± 4.94	0.86
**Clinical parameters in survivors**			
Age at diagnosis (years)	6.96 ± 3.56		
Duration since completion of therapy (years)	13.71 ± 7.63		
Cumulative anthracycline dose (mg/m^2^)	200.7 ± 126.80		
With dose exceeding 250 mg/m^2^ (#)	6 (30%)		
Use of Dexrazoxane (#)	1 (5%)		
Use of radiation therapy (#)	5 (25%)		
Thoracic (mantle, lung, paraaortic) (#)	3 (15%)		
Abdominal (whole, spleen, liver) (#)	3 (15%)		
Cranial (#)	1 (5%)		
Pelvic (#)	1 (5%)		
**Diagnosis**			
Acute Lymphocytic Leukemia (ALL) (#)	5 (25%)		
Ewing's Sarcoma (#)	3 (15%)		
Hodgkin's Lymphoma (#)	3 (15%)		
Non-Hodgkin's Lymphoma (#)	3 (15%)		
Acute Myeloid Leukemia (AML) (#)	2 (10%)		
Osteosarcoma (#)	1 (5%)		
Rhabdomyosarcoma (#)	1 (5%)		
Wilms Tumor (#)	1 (5%)		
**Blood biomarkers**			
Total cholesterol	4.45 ± 0.92	4.53 ± 0.84	0.78
Triglycerides	1.00 ± 0.45	0.82 ± 0.33	0.19
LDLc	2.38 ± 0.83	2.56 ± 0.81	0.52
HDLc	1.61 ± 0.38	1.60 ± 0.32	0.91
hs-CRP	2.95 ± 5.81	0.94 ± 1.23	0.19
Fasting glucose	4.88 ± 0.91	5.08 ± 0.49	0.44
Insulin	61.82 ± 25.61	60.61 ± 31.90	0.90
Hb A1c	5.00 ± 0.27	4.99 ± 0.24	0.95
**Vascular parameters**			
IMT (cm)	0.05 ± 0.006	0.04 ± 0.03	0.59

Values reported as average +/− standard deviation, or number of participants (#).

**Table 2:** Cardiovascular measurements during rest and exercise.

Parameter	Cancer survivor Avg ± SD	Control Avg ± SD	*p*
Rest SBP (mmHg)	118.5 ± 11.0	118.52 ± 10.27	0.99
Rest DBP (mmHg)	68.1 ± 6.8	71.86 ± 8.32	0.27
Rest HR (bpm)	71.4 ± 8.7	67.00 ± 12.06	0.17
Rest max diameter (cm)	0.61 ± 0.05	0.62 ± 0.07	0.60
Rest min diameter (cm)	0.53 ± 0.05	0.54 ± 0.06	0.51
Rest distensibility by US	0.14 ± 0.03	0.14 ± 0.03	0.77
Rest tonometry PWV (m/s)	6.45 ± 0.97	7.02 ± 1.01	0.08
Rest UUI PWV-SF (m/s)	6.65 ± 0.94	6.87 ± 1.16	0.56
Rest UUI PWV-DN (m/s)	8.26 ± 1.52	8.63 ± 1.39	0.50
Exercise SBP (mmHg)	143.53 ± 20.15	144.05 ± 15.44	0.56
Exercise DBP (mmHg)	80.47 ± 13.06	79 ± 8.92	0.42
Exercise HR (bpm)	120.42 ± 15.40	114.70 ± 17.38	0.30
Exercise max diameter (cm)	0.64 ± 0.07	0.65 ± 0.09	0.56
Exercise min diameter (cm)	0.53 ± 0.05	0.55 ± 0.07	0.42
Exercise distensibility by US	0.17 ± 0.05	0.16 ± 0.03	0.66
Exercise tonometry PWV (m/s)	7.22 ± 1.28	8.13 ± 1.41	**0.04**
Exercise UUI PWV-SF (m/s)	7.95 ± 0.93	8.31 ± 1.57	0.46
Exercise UUI PWV-DN (m/s)	9.50 ± 1.19	9.66 ± 1.29	0.76

### PWV measurements by UUI

**Figure 3:**
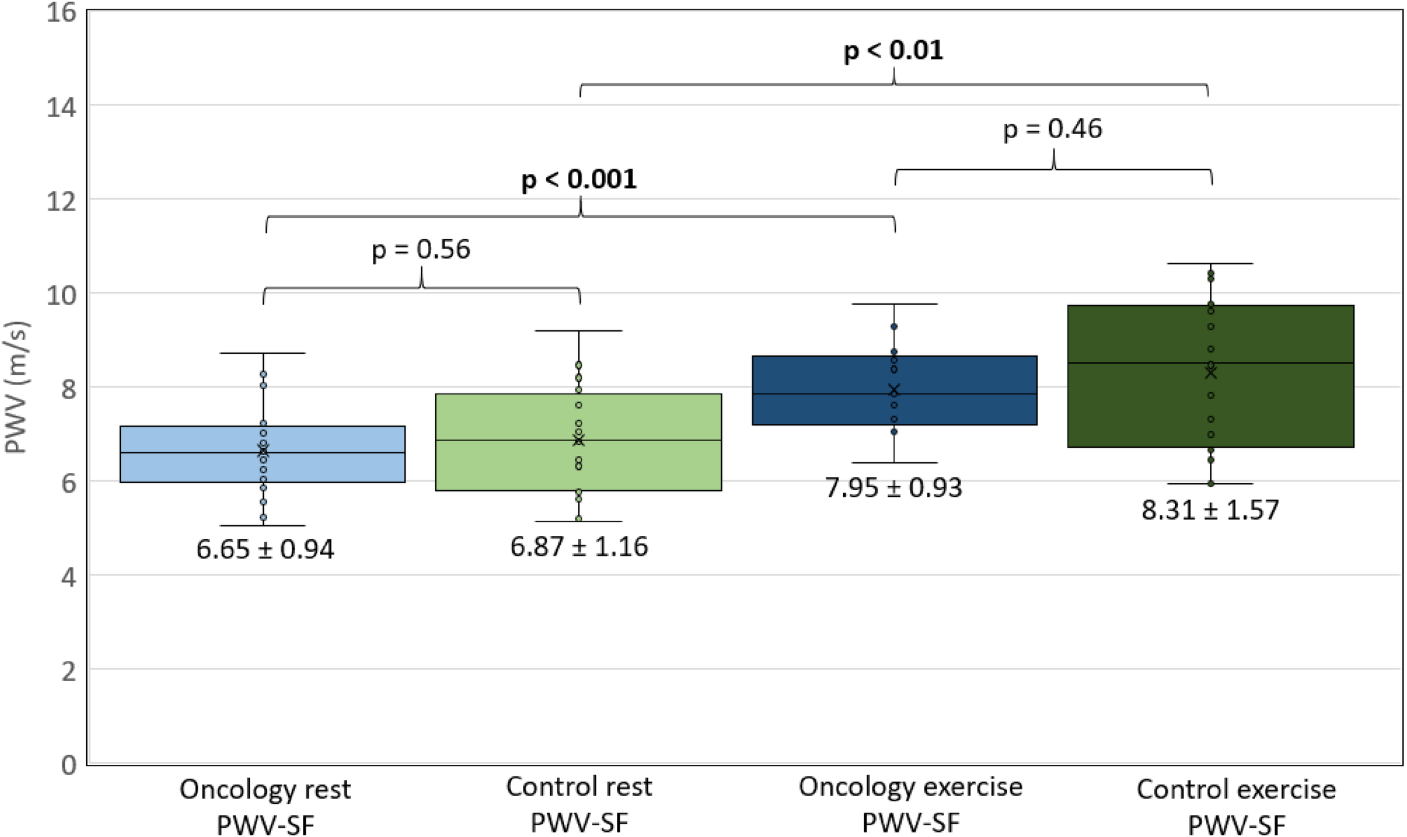
PWV-SF comparisons. Measurements of pulse wave velocity at the systolic foot, as measured with UUI, represented on a box and whisker plot. *X*-axis defines group (oncology or control) and condition (rest or exercise). *Y*-axis represents PWV (m/s). Mean PWV +/− SD listed under each box. *p* < 0.05 considered significant, and represented in bold.

Figure 3 shows PWV-SF measurements in both groups, at rest and exercise. PWV-SF was significantly different at rest vs. exercise condition in the CCS group (*p* < 0.001) and HV group (*p* < 0.01). However, there was no difference in PWV-SF between CCS and HV groups at rest (*p* = 0.56) or during exercise (*p* = 0.46).

**Figure 4:**
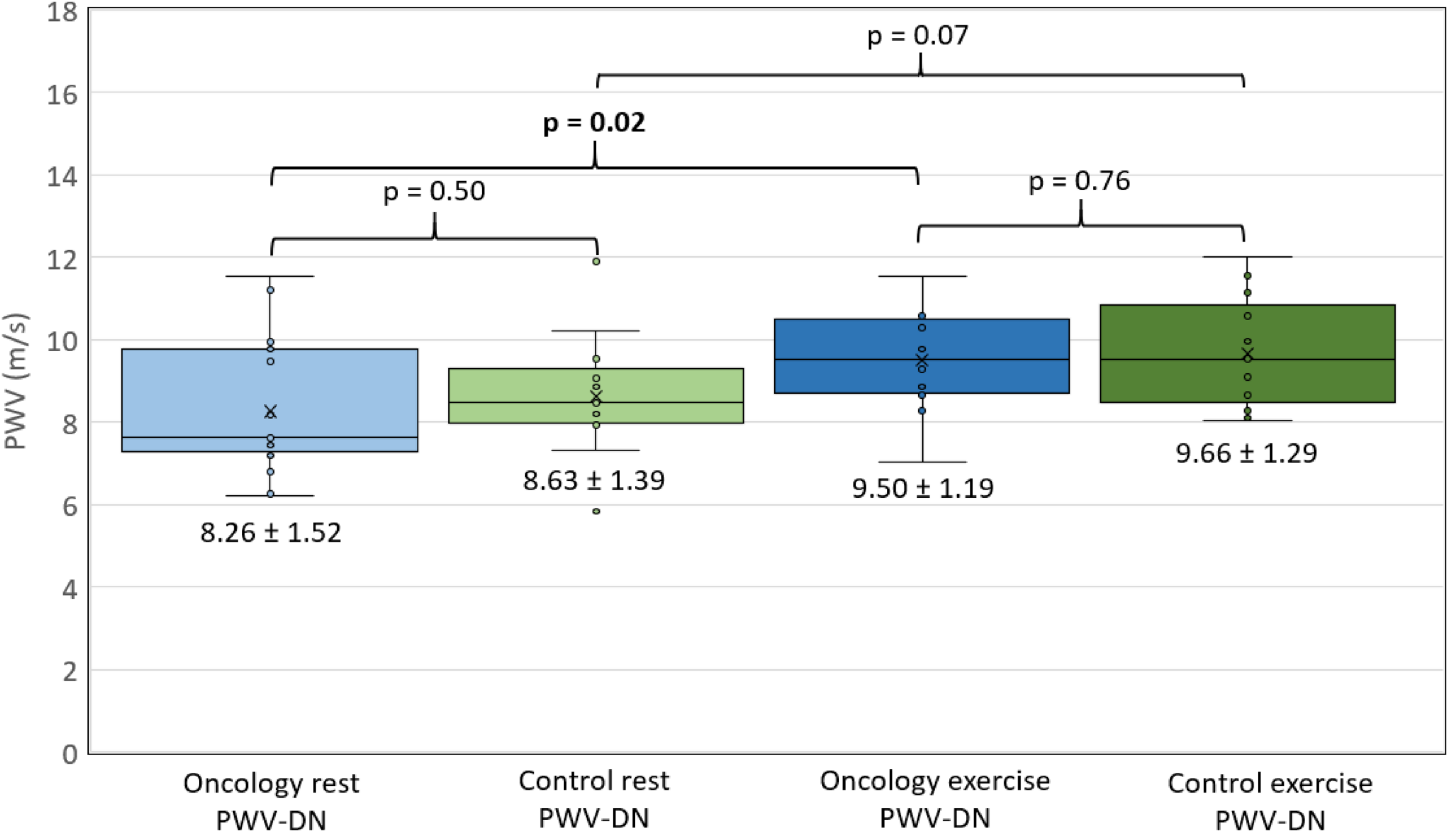
PWV-DN comparisons. Measurements of pulse wave velocity at the dicrotic notch, as measured with UUI, represented on a box and whisker plot. *X*-axis defines group (oncology or control) and condition (rest or exercise). *Y*-axis represents PWV (m/s). Mean PWV +/− SD listed under each box. *p* < 0.05 considered significant, and represented in bold.

Figure 4 shows PWV-DN measurements in both groups, at rest and exercise. PWV-DN was significantly different at rest vs. during exercise in the CCS group (*p* = 0.02), but not in the HV group (*p* = 0.07). There was no difference in PWV-DN between CCS and HV groups at rest (*p* = 0.50) or during exercise (*p* = 0.76).

### PWV correlations with other parameters at rest

Table 3 outlines the correlations between PWV and other clinical and cardiovascular parameters at rest in CCS and HV. There was a significant positive correlation between PWV-SF and age in both groups. In the CCS group, PWV-SF also correlated with total cholesterol and LDLc. In the HV group, PWV-SF also correlated with weight (*p* = 0.007), BMI (*p* = 0.017), BP (*p* = 0.005 for DBP, *p* = 0.014 for SBP), IMT (*p* = 0.039), and systolic and diastolic carotid diameter (*p* < 0.001 for both).

**Table 3:** Correlations between PWVs and other parameters at rest.

Parameters	PWV-SF				PWV-DN			
	Cancer survivor		Control		Cancer survivor		Control	
	*r*	*p*	*r*	*p*	*r*	*p*	*r*	*p*
Age	0.522	**0**.**044**	0.600	**0**.**014**	0.139	0.569	0.551	0.051
Weight	0.166	0.485	0.667	**0**.**007**	0.121	0.622	0.343	0.275
Height	0.298	0.201	0.457	0.457	0.098	0.690	0.406	0.169
BMI	0.045	0.85	0.606	**0**.**017**	0.092	0.708	0.186	0.563
IMT	−0.453	0.103	0.536	**0**.**039**	−0.152	0.619	0.127	0.678
Tonometry PWV	0.679	**<0**.**005**	0.752	**0**.**001**	0.291	0.242	0.692	**0**.**009**
UUI PWV-SF	–	–	–	–	0.673	**0**.**002**	0.796	**0**.**001**
UUI PWV-DN	0.673	**<0**.**005**	0.796	**0**.**001**		** **		** **
DBP	0.297	0.203	0.661	**0**.**005**	−0.191	0.435	0.524	0.066
SBP	−0.202	0.393	0.599	**0**.**014**	−0.388	0.101	0.336	0.262
HR	−0.167	0.480	0.441	0.087	−0.002	0.994	0.095	0.757
Max carotid diameter	−0.361	0.117	0.769	**<0**.**001**	0.025	0.919	0.452	0.121
Min carotid diameter	−0.213	0.367	0.826	**<0**.**001**	0.097	0.692	0.634	**0**.**020**
β-index	0.093	0.696	0.400	0.124	0.171	0.483	0.632	**0**.**020**
Age at diagnosis	0.249	0.304	NA	NA	0.148	0.557	NA	NA
Duration since therapy completion	0.394	0.095	NA	NA	0.089	0.727	NA	NA
Cumulative anthracycline dose	−0.175	0.473	NA	NA	−0.185	0.463	NA	NA
Cumulative dose of Radiation	−0.944	0.056	NA	NA	−0.796	0.204	NA	NA
Total cholesterol	0.571	**0**.**021**	0.160	0.601	0.429	0.111	−0.046	0.892
Triglycerides	−0.269	0.313	0.442	0.130	−0.008	0.979	−0.013	0.970
LDLc	0.651	**0**.**006**	0.112	0.716	0.514	0.051	−0.171	0.615
HDLc	0.108	0.691	−0.114	0.710	−0.097	0.730	0.268	0.426
hs-CRP	0.195	0.469	0.564	**0**.**045**	0.330	0.230	0.116	0.734
Fasting glucose	0.374	0.154	0.488	0.091	0.275	0.321	0.521	0.100
Insulin	−0.223	0.407	0.217	0.476	−0.299	0.279	−0.028	0.936
Hb A1c	0.243	0.364	−0.324	0.281	0.216	0.439	0.091	0.789

PWV-DN in the CCS group had no significant correlations. In the HV group, PWV-DN correlated to minimum (diastolic) carotid diameter (*p* = 0.020) and B-index (*p* = 0.020).

Multiple regression was conducted for the correlated factors described above at rest. In the CCS group, age (*p* = 0.024) and LDL-c (*p* = 0.019) remained significantly correlated to PWV-SF. In the HV group, age (*p* = 0.048), SBP and DBP carotid diameter (*p* = 0.045 and *p* = 0.010, respectively) remained significantly correlated to PWV-SF.

### Adaptation to cardiovascular stress

Table 4 outlines correlations between PWV and other clinical and cardiovascular parameters during exercise in HV and CCS. During exercise, PWV-SF correlates significantly with age, and max (systolic) carotid diameter in both groups. In the HV group, PWV-SF was correlated with weight (*p* = 0.016), height (*p* = 0.025), IMT (*p* = 0.001), and min (diastolic) carotid diameter (*p* = 0.015). PWV-SF in the CCS group additionally correlated with B-index (*p* = 0.031), and LDLc (*p* = 0.031).

**Table 4:** Correlations between PWV and other parameters during exercise.

Parameters	PWV-SF				PWV-DN			
	Cancer survivor		Control		Cancer survivor		Control	
	*r*	*p*	*r*	*p*	*r*	*p*	*r*	*p*
Age	0.652	**0.016**	0.513	**0.042**	0.127	0.695	0.464	0.110
Weight	−0.169	0.582	0.611	**0.016**	−0.190	0.554	0.708	**0.010**
Height	0.235	0.440	0.556	**0.025**	−0.320	0.310	0.685	**0.010**
BMI	−0.358	0.230	0.492	0.063	−0.068	0.833	0.488	0.108
IMT	−0.644	0.847	0.647	**0.006**	−0.090	0.847	0.263	0.385
Tonometry PWV	0.652	**0.016**	0.748	**0.001**	0.224	0.483	0.793	**0.001**
UUI PWV-SF	–	–	–	–	0.561	**0.048**	0.767	**0.002**
UUI PWV-DN	0.561	0.058	0.767	**0.002**	–	–	–	–
DBP	−0.039	0.230	0.460	0.073	−0.258	0.418	0.305	0.311
SBP	−0.064	0.835	0.190	0.482	−0.323	0.306	0.271	0.371
HR	−0.316	0.293	−0.094	0.730	−0.313	0.322	−0.386	0.193
Max carotid diameter	−0.719	**0.008**	0.580	**0.018**	−0.621	**0.041**	0.629	**0.021**
Min carotid diameter	−0.427	0.167	0.595	**0.015**	−0.451	0.164	0.655	**0.015**
β-index	0.622	**0.031**	0.098	0.729	0.437	0.178	0.252	0.406
Age at diagnosis	0.049	0.881	NA	NA	0.044	0.897	NA	NA
Duration since therapy completion	0.544	0.064	NA	NA	0.073	0.831	NA	NA
Cumulative anthracycline dose	−0.167	0.603	NA	NA	−0.113	0.740	NA	NA
Cumulative dose of Radiation	–	–	NA	NA	–	–	NA	NA
Total cholesterol	0.524	0.080	−0.017	0.956	−0.045	0.897	−0.344	0.230
Triglycerides	−0.431	0.162	0.330	0.271	−0.423	0.194	−0.214	0.528
LDLc	0.621	**0.031**	0.005	0.987	0.126	0.711	−0.483	0.132
HDLc	0.210	0.511	−0.207	0.497	−0.205	0.545	0.404	0.218
hs-CRP	−0.128	0.691	0.520	0.069	0.009	0.978	0.280	0.405
Fasting glucose	0.051	0.874	0.362	0.224	−0.175	0.606	0.354	0.285
Insulin	−0.396	0.203	0.150	0.626	−0.235	0.487	−0.069	0.841
Hb A1c	0.143	0.657	−0.402	0.174	0.230	0.496	−0.347	0.299

During exercise, PWV-DN correlated with max (systolic) carotid diameter in both groups. PWV-DN in the HV group additionally correlated with weight (*p* = 0.010), height (*p* = 0.010) and diastolic carotid diameter (*p* = 0.015).

Table 5 outlines the change in SBP, DBP, pulse pressure (PP), heart rate (HR), PWV-SF and PWV-DN between rest and exercise in both groups. There was a significant difference between the change in heart rate in CCS and HV (*p* < 0.01), but not in other parameters.

**Table 5:** Exercise adaptation.

Parameter	Cancer survivor *n* = 20	Control *n* = 21	*p*
ΔSBP (mmHg)	24.26 ± 17.8	25.35 ± 14.97	0.84
ΔDBP (mmHg)	10.58 ± 13.60	6.85 ± 8.66	0.33
ΔPP (mmHg)	13.68 ± 17.78	18.50 ± 9.64	0.32
ΔHR (bpm)	49.16 ± 10.59	35.70 ± 14.98	**<0**.**01**
ΔPWV-SF (m/s)	1.25 ± 0.33	1.45 ± 0.59	0.30
ΔPWV-DN (m/s)	1.42 ± 0.99	1.03 ± 0.79	0.31

Change in parameters between rest and submaximal exercise. Values reported as mean ± SD.

### Intra- and inter-observer variability

**Figure 5:**
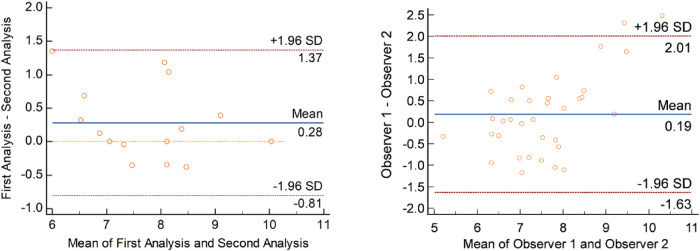
Intra-observer (left) and inter-observer (right) variability. Bland-Altman plots representing inter- and intra-observer variability. Each point represents one PWV measurement. Left panel demonstrates intra-observer analysis. Measured as repeated analysis − original analysis. Right panel demonstrated inter-observer analysis. Measured as Observer 2 − Observer 1.

Regarding the intra-observer variability, 15 pulse waves (8 PWV-SF, 7 PWV-DN) obtained by UUI were re-analyzed and are depicted in Figure 5 as a Bland-Altman plot. No significant bias was observed, with a mean difference of 0.28 m/s, and limits of agreement between 1.37 and −0.81 m/s.

Regarding the inter-observer variability, 34 pulse waves were re-analyzed (12 systolic foot, 12 dicrotic notch), and are depicted in Figure 5 as a Bland-Altman plot. No significant bias was observed, with a mean difference of 0.19 m/s, and limits of agreement between 2.01 and −1.63 m/s.

## Discussion

In this study, UUI measurement of local left carotid PWV was used to assess arterial stiffness in CCS treated with anthracyclines both at rest and at the end of submaximal exercise study. Overall, we observed no significant differences in PWV measurements between CCS and healthy controls either at rest or after exercise. However, the correlation between PWV and certain factors did differ between the two groups. Importantly, PWV-SF in CCS was not correlated with BMI, IMT, BP, and carotid diameter, all of which have been shown to be correlated to the PWV of healthy individuals in previous studies (23–27). This may indicate that, while there was overall no difference between the two matched cohorts at large, that different factors are involved in the mechanism of arterial stiffening. Multiple regression showed that age and LDL-c were independent and significant contributors to PWV-SF in CCS. These factors are not directly attributable to cancer treatment and are not novel findings; age is a known primary contributor to arterial stiffness, as well as high LDL-c and atherosclerosis (28–30). Multiple regression for HV showed that age and carotid diameter are independent and significant contributors to PWV-SF in the control group. Carotid diameter is a parameter commonly used to assess local arterial stiffness, such as in the Bramwell-Hill equation (31). This data may suggest that, while there is not an overall difference in the net arterial stiffness between CCS and healthy volunteers, there may be different physiological contributors to arterial stiffness between the two groups.

### Global vs. local stiffness

We estimated both global (by tonometry) and local (by UUI) PWV. We observed no significant difference between local PWV-SF measurements of the left common carotid artery and global carotid-femoral tonometry measurements in either group. Based on the hypothesized mechanism of vascular injury by anthracyclines leading to endothelial dysfunction, there is no evidence that any particular arteries will be targeted, but it is reasonable to hypothesize that arteries with high dependence on endothelial control may show more dysfunction (4). In fact, there is little research about the local PWV of different arteries throughout the body, and UUI has, to date, primarily been published with use on the carotid artery (14, 25, 32–40). Therefore, the significance of absence of difference between global and left carotid artery estimates is unclear. It is also unclear whether results would vary with the analysis of different arteries, specifically peripheral arteries. Chow et al. showed increased brachial artery stiffness (estimated by B-stiffness or tonometry related calculations) in a CCS group treated with anthracyclines (41). This should be one area of investigation in future UUI studies.

### Acute vs. chronic anthracycline effects

This study analyzed the vascular effects of anthracyclines exclusively in the chronic setting, with an average of 13.71 ± 7.63 years since therapy completion. Cardiotoxicity due to anthracyclines is typically classified as acute/early (effects during treatment), subacute (within a year of treatment), or chronic (over a year after exposure) (42). Significant increases in vascular stiffness have been shown in the acute phase, which was not studied in our population (43). Existing literature regarding vascular stiffness has studied patients after an average of 2 years, 7 years, and 10 years after anthracycline treatment (44–46). Among these, the 2 and 10-year follow up studies both showed a difference between anthracycline treated and healthy control groups, but the 7-year follow up did not. This discrepancy points to a future area of study. In particular, the more chronic effects after multiple decades have not yet been studied in the context of vascular changes. In the context of cardiac toxicity, large cross sectional studies following some patients over 30 years after treatment has shown increased subclinical and clinical cardiac toxicity (6).

### Vascular vs. cardiac anthracycline toxicity

The cardiac toxicity of anthracyclines is much better studied and understood compared to vascular toxicity. While the exact mechanism continues to be studied, it is thought that the mechanism of cardiac toxicity involves the activation of cell death pathways and inhibition of mitochondrial biogenesis (4). Specifically, left ventricular dysfunction long-term after anthracycline treatment is well documented (47–49). It is important to note that left ventricular function is hemodynamically dependant on the systemic arterial system. This is represented in the form of afterload (50, 51). Studies of breast cancer patients treated with anthracyclines have shown that an increase in arterial elastance leads to abnormal ventricular-arterial coupling, which is a predictor of cardiac dysfunction related to anthracyclines (52). UUI has emerging use as a tool for measuring myocardial stiffness (13). Measurement of both myocardial and arterial stiffness by UUI in future studies would be useful to further elucidate this mechanism and interpret our current results. Additionally, it is well known that a cumulative anthracycline dose >250 mg/m^2^ is related to a significantly increased risk of cardiac disease (6). The average cumulative anthracycline dose in our CCS population was 200.7 ± 126.80 mg/m^2^, with only 6 CCS participants (30%) having a cumulative dose exceeding 250 mg/m^2^. As such, it is possible that the threshold for high risk of cardiotoxicity is translated to vascular toxicity.

### Cardiovascular stress by exercise

This study included an exercise component to induce cardiovascular stress. Previous studies have suggested that vascular toxicity may be attributable to endothelial dysfunction (53). Endothelial dysfunction is an impairment of the inner lining of all blood vessels that controls vasodilation and vasoconstriction. Since exercise is a cardiovascular stress, vasodilation and vasoconstriction of arteries around the body are induced (54). Exercise induced stress has previously been shown to increase PWV in individuals with coronary artery disease (55). Additionally, PWV should increase with exercise along with BP through the pressure dependence of arterial stiffness (56). This has been shown in healthy populations (31). Li et al. observed vascular stiffness post-anthracycline treatment at rest and exercise (57). However, as opposed to this current study, stiffness was estimated by applanation tonometry and radial augmentation index. Additionally, their CCS study population was older at 25.0 ± 5.9 years and had a longer duration since the completion of anthracycline therapy at 15.4 ± 5.9 years. Cumulative anthracycline doses were also higher at 258 ± 110 mg/m^2^. Cifra et al. demonstrated dynamic myocardial response to exercise in a similar CCS population, and demonstrated a similar response in HR and BP (i.e., no difference between CCS and HV) as in this current study (58). As such, the potential impact of anthracyclines on cardiovascular adaptation to stress should continue to be explored in future studies, especially due to its relationship with left ventricular dysfunction as explained above.

### Limitations

There are several limitations to this study. Primarily, the small population size of 41 participants limits the statistical power. This was due to the relatively new and involved method of measuring arterial stiffness with UUI, which is not yet clinically available. The small sample size meant that we were not able to statistically analyze categorical data, including radiation data, which can be an important contributor to cardiovascular toxicity. Similarly, multivariate analysis was limited due to the small sample size. Additionally, there were limitations in the exercise trial. To ensure images were captured before the participant stopped exercising, ultrafast acquisitions (and the other coinciding measurements) were taken during stage 3 of the Bruce protocol (75 Watts) for all patients. This stage was at different levels of personal exertion for each participant. Finally, this was a cross-sectional study which evaluated participants at a single point in time. As mentioned in the discussion, longer-term effects of anthracyclines and long-term follow up are not known for vascular toxicity. Ideally, participants could have been tracked multiple times over several years to determine the trajectory of vascular toxicity.

## Conclusion

Overall, there were no significant differences found between left common carotid stiffness measured by UUI in HV and CCS populations in our study. This continued to be true under cardiovascular stress, induced by an exercise trial. While there is a documented risk of cardiotoxicity with anthracycline treatment, this study does not provide evidence that anthracyclines alter vascular properties in a CCS population treated with an average dose <250 mg/m^2^. The use of UUI for studying the impact of anthracyclines in arterial stiffness is an interesting and emerging field of research. As such, additional studies with large cohorts are needed to confirm our observations.

## Data Availability

The raw data supporting the conclusions of this article will be made available by the authors, without undue reservation.

## References

[1] Government of Canada SC. Trends in paediatric cancer survival in Canada, 1992 to 2017 (2021). Available at: https://www150.statcan.gc.ca/n1/pub/82-003-x/2021002/article/00001-eng.htm (Cited June 30, 2022).

[2] Volkova M Russell R. Anthracycline cardiotoxicity: prevalence, pathogenesis and treatment. Curr Cardiol Rev. (2011) 7(4):214–20. 10.2174/157340311799960645 22758622 PMC3322439

[3] Khouri MG Douglas PS Mackey JR Martin M Scott JM Scherrer-Crosbie M Cancer therapy-induced cardiac toxicity in early breast cancer: addressing the unresolved issues. Circulation. (2012) 126(23):2749–63. 10.1161/CIRCULATIONAHA.112.100560 23212997 PMC3667651

[4] Henriksen PA. Anthracycline cardiotoxicity: an update on mechanisms, monitoring and prevention. Heart. (2018) 104(12):971–7. 10.1136/heartjnl-2017-312103 29217634

[5] Kremer LC van Dalen EC Offringa M Ottenkamp J Voûte PA. Anthracycline-induced clinical heart failure in a cohort of 607 children: long-term follow-up study. J Clin Oncol. (2001) 19(1):191–6. 10.1200/JCO.2001.19.1.191 11134212

[6] Mulrooney DA Yeazel MW Kawashima T Mertens AC Mitby P Stovall M Cardiac outcomes in a cohort of adult survivors of childhood and adolescent cancer: retrospective analysis of the childhood cancer survivor study cohort. Br Med J. (2009) 339:b4606. 10.1136/bmj.b4606 19996459 PMC3266843

[7] Grenier MA Lipshultz SE. Epidemiology of anthracycline cardiotoxicity in children and adults. Semin Oncol. (1998) 25(4 Suppl 10):72–85. 9768828

[8] Abu-Khalaf MM Juneja V Chung GG DiGiovanna MP Sipples R McGurk M Long-term assessment of cardiac function after dose-dense and -intense sequential doxorubicin (A), paclitaxel (T), and cyclophosphamide (C) as adjuvant therapy for high risk breast cancer. Breast Cancer Res Treat. (2007) 104(3):341–9. 10.1007/s10549-006-9413-7 17051423

[9] Lipshultz SE Franco VI Miller TL Colan SD Sallan SE. Cardiovascular disease in adult survivors of childhood cancer. Annu Rev Med. (2015) 66:161–76. 10.1146/annurev-med-070213-054849 25587648 PMC5057395

[10] Parr SK Liang J Schadler KL Gilchrist SC Steele CC Ade CJ. Anticancer therapy–related increases in arterial stiffness: a systematic review and meta-analysis. J Am Heart Assoc. (2020) 9(14):e015598. 10.1161/JAHA.119.015598 32648507 PMC7660726

[11] Bérard E Bongard V Ruidavets JB Amar J Ferrières J. Pulse wave velocity, pulse pressure and number of carotid or femoral plaques improve prediction of cardiovascular death in a population at low risk. J Hum Hypertens. (2013) 27(9):529–34. 10.1038/jhh.2013.8 23426066

[12] Segers P Rietzschel ER Chirinos JA. How to measure arterial stiffness in humans. Arterioscler Thromb Vasc Biol. (2020) 40(5):1034–43. 10.1161/ATVBAHA.119.313132 31875700 PMC7180118

[13] Villemain O Baranger J Friedberg MK Papadacci C Dizeux A Messas E Ultrafast ultrasound imaging in pediatric and adult cardiology: techniques, applications, and perspectives. JACC Cardiovasc Imaging. (2020) 13(8):1771–91. 10.1016/j.jcmg.2019.09.019 31734211

[14] Yin LX Ma CY Wang S Wang YH Meng PP Pan XF Reference values of carotid ultrafast pulse-wave velocity: a prospective, multicenter, population-based study. J Am Soc Echocardiogr. (2021) 34(6):629–41. 10.1016/j.echo.2021.01.003 33422666

[15] Couade M Pernot M Messas E Emmerich J Hagège A Fink M Ultrafast imaging of the arterial pulse wave. IRBM. (2011) 32(2):106–8. 10.1016/j.irbm.2011.01.012

[16] Muntner P Shimbo D Carey RM Charleston JB Gaillard T Misra S Measurement of blood pressure in humans: a scientific statement from the American heart association. Hypertension. (2019) 73(5):e35–66. 10.1161/HYP.0000000000000087 30827125 PMC11409525

[17] Mattoo TK. Definition and diagnosis of hypertension in children and adolescents (2021). Available at: https://www.uptodate.com/contents/definition-and-diagnosis-of-hypertension-in-children-and-adolescents?source=autocomplete&index=1∼4=h&searchypertension%20.

[18] Bruce RA Lovejoy FW Pearson R Yu PNG Brothers GB Velasquez T. Normal respiratory and circulatory pathways of adaptation in exercise 1. J Clin Invest. (1949) 28(6 Pt 2):1423–30. 10.1172/JCI102207 15407661 PMC439698

[19] Sarkola T Manlhiot C Slorach C Bradley TJ Hui W Mertens L Evolution of the arterial structure and function from infancy to adolescence is related to anthropometric and blood pressure changes. Arterioscler Thromb Vasc Biol. (2012) 32(10):2516–24. 10.1161/ATVBAHA.112.252114 22837471

[20] Cooke AB Kuate Defo A Dasgupta K Papaioannou TG Lee J Morin SN Methodological considerations for the measurement of arterial stiffness using applanation tonometry. J Hypertens. (2021) 39(3):428–36. 10.1097/HJH.0000000000002665 33031179

[21] Loupas T Powers JT Gill RW. An axial velocity estimator for ultrasound blood flow imaging, based on a full evaluation of the Doppler equation by means of a two-dimensional autocorrelation approach. IEEE Trans Ultrason Ferroelectr Freq Control. (1995) 42(4):672–88. 10.1109/58.393110

[22] Vos HJ van Dalen BM Heinonen I Bosch JG Sorop O Duncker DJ Cardiac shear wave velocity detection in the porcine heart. Ultrasound Med Biol. (2017) 43(4):753–64. 10.1016/j.ultrasmedbio.2016.11.015 28065540

[23] The Reference Values for Arterial Stiffness’ Collaboration. Determinants of pulse wave velocity in healthy people and in the presence of cardiovascular risk factors: ‘establishing normal and reference values’. Eur Heart J. (2010) 31(19):2338–50. 10.1093/eurheartj/ehq165 20530030 PMC2948201

[24] Kim EJ Park CG Park JS Suh SY Choi CU Kim JW Relationship between blood pressure parameters and pulse wave velocity in normotensive and hypertensive subjects: invasive study. J Hum Hypertens. (2007) 21(2):141–8. 10.1038/sj.jhh.1002120 17136108

[25] Pan FS Xu M Yu L Luo J Li MY Liang JY Relationship between carotid intima-media thickness and carotid artery stiffness assessed by ultrafast ultrasound imaging in patients with type 2 diabetes. Eur J Radiol. (2019) 1:34–40. 10.1016/j.ejrad.2018.12.016 30691662

[26] Ma Y Choi J Hourlier-Fargette A Xue Y Chung HU Lee JY Relation between blood pressure and pulse wave velocity for human arteries. Proc Natl Acad Sci. (2018) 115(44):11144–9. 10.1073/pnas.1814392115 30322935 PMC6217416

[27] Bailey MA Davies JM Griffin KJ Bridge KI Johnson AB Sohrabi S Carotid-femoral pulse wave velocity is negatively correlated with aortic diameter. Hypertens Res. (2014) 37(10):926–32. 10.1038/hr.2014.101 24919482

[28] Lee HY Oh BH. Aging and arterial stiffness. Circ J Off J Jpn Circ Soc. (2010) 74(11):2257–62. 20962429 10.1253/circj.cj-10-0910

[29] Wilkinson I Cockcroft JR. Cholesterol, lipids and arterial stiffness. Adv Cardiol. (2007) 44:261–77. 10.1159/000096747 17075215

[30] Ference BA Ginsberg HN Graham I Ray KK Packard CJ Bruckert E Low-density lipoproteins cause atherosclerotic cardiovascular disease. 1. Evidence from genetic, epidemiologic, and clinical studies. A consensus statement from the European atherosclerosis society consensus panel. Eur Heart J. (2017) 38(32):2459–72. 10.1093/eurheartj/ehx144 28444290 PMC5837225

[31] Rasouli R Baranger J Slorach C Nguyen M Segers P Guerra V Local arterial stiffness assessment: comparison of pulse wave velocity assessed by ultrafast ultrasound imaging versus the bramwell-hill equation. J Am Soc Echocardiogr. (2022) 35(11):1185–8. 10.1016/j.echo.2022.07.011 35863547

[32] Wang Y Zhu Z Ma X Liu W Jiang X Wu Y Individualized references of carotid stiffening quantified with ultrafast ultrasound imaging: model construction and preliminary validation. Ultrasound Med Biol. (2022) 48(8):1528–36. 10.1016/j.ultrasmedbio.2022.03.017 35595590

[33] Timóteo AT. Carotid pulse wave velocity by ultrafast ultrasound: a step forward for noninvasive assessment of diabetic patients. J Clin Ultrasound. (2022) 50(3):317–8. 10.1002/jcu.23154 35277981

[34] An X Li Y Shi S Ge L Li Y. Clinical significance and influencing factors of carotid pulse wave velocity in patients with diabetic microangiopathy. J Clin Ultrasound. (2022) 50(3):309–16. 10.1002/jcu.23153 35150445

[35] Zhu ZQ Chen LS Jiang XZ Wu YY Zou C Luan Y Absent atherosclerotic risk factors are associated with carotid stiffening quantified with ultrafast ultrasound imaging. Eur Radiol. (2021) 31(5):3195–206. 10.1007/s00330-020-07405-4 33068187

[36] Yang W Wang Y Yu Y Mu L Kong F Yang J Establishing normal reference value of carotid ultrafast pulse wave velocity and evaluating changes on coronary slow flow. Int J Cardiovasc Imaging. (2020) 36(10):1931–9. 10.1007/s10554-020-01908-3 32506285

[37] Li Y Zhang Y Geng X Zhao S Sun YX Wang YB. Increased carotid stiffness detected by ultrafast ultrasound imaging is associated with the gensini score. Med Ultrason. (2020) 22(2):183–8. 10.11152/mu-2323 32399526

[38] Zhu ZQ Chen LS Wang H Liu FM Luan Y Wu LL Carotid stiffness and atherosclerotic risk: non-invasive quantification with ultrafast ultrasound pulse wave velocity. Eur Radiol. (2019) 29(3):1507–17. 10.1007/s00330-018-5705-7 30187117

[39] Li X Jiang J Zhang H Wang H Han D Zhou Q Measurement of carotid pulse wave velocity using ultrafast ultrasound imaging in hypertensive patients. J Med Ultrason. (2017) 44(2):183–90. 10.1007/s10396-016-0755-4 27933439

[40] Mirault T Pernot M Frank M Couade M Niarra R Azizi M Carotid stiffness change over the cardiac cycle by ultrafast ultrasound imaging in healthy volunteers and vascular ehlers-danlos syndrome. J Hypertens. (2015) 33(9):1890–6. 10.1097/HJH.0000000000000617 26248323

[41] Chow AY Chin C Dahl G Rosenthal DN. Anthracyclines cause endothelial injury in pediatric cancer patients: a pilot study. J Clin Oncol. (2006) 24(6):925–8. 10.1200/JCO.2005.03.5956 16484703

[42] Clinical manifestations, monitoring, and diagnosis of anthracycline-induced cardiotoxicity. Available at: https://www.uptodate.com/contents/clinical-manifestations-monitoring-and-diagnosis-of-anthracycline-induced-cardiotoxicity?search=anthracycline%20cardiotoxicity&source=search_result&selectedTitle=1∼150&usage_type=default&display_rank=1 (Cited September 27, 2022).

[43] Drafts BC Twomley KM D’Agostino R Lawrence J Avis N Ellis LR Low to moderate dose anthracycline-based chemotherapy is associated with early noninvasive imaging evidence of subclinical cardiovascular disease. JACC Cardiovasc Imaging. (2013) 6(8):877–85. 10.1016/j.jcmg.2012.11.017 23643285 PMC3745801

[44] Herceg-Cavrak V Ahel V Batinica M Matec L Kardos D. Increased arterial stiffness in children treated with anthracyclines for malignant disease. Coll Antropol. (2011) 35(2):389–95. 21755708

[45] Krystal JI Reppucci M Mayr T Fish JD Sethna C. Arterial stiffness in childhood cancer survivors. Pediatr Blood Cancer. (2015) 62(10):1832–7. 10.1002/pbc.25547 25895119

[46] Yersal Ö Eryilmaz U Akdam H Meydan N Barutca S. Arterial stiffness in breast cancer patients treated with anthracycline and trastuzumab-based regimens. Cardiol Res Pract. (2018) 2018:5352914. 10.1155/2018/5352914 29854434 PMC5954934

[47] Lipshultz SE Lipsitz SR Sallan SE Dalton VM Mone SM Gelber RD Chronic progressive cardiac dysfunction years after doxorubicin therapy for childhood acute lymphoblastic leukemia. J Clin Oncol. (2005) 23(12):2629–36. 10.1200/JCO.2005.12.121 15837978

[48] Ganame J Claus P Uyttebroeck A Renard M D’hooge J Bijnens B Myocardial dysfunction late after low-dose anthracycline treatment in asymptomatic pediatric patients. J Am Soc Echocardiogr. (2007) 20(12):1351–8. 10.1016/j.echo.2007.04.007 17604960

[49] Cheung YF Hong WJ Chan GC Wong SJ Ha SY. Left ventricular myocardial deformation and mechanical dyssynchrony in children with normal ventricular shortening fraction after anthracycline therapy. Heart Br Card Soc. (2010) 96(14):1137–41. 10.1136/hrt.2010.194118 20511624

[50] Kass DA Kelly RP. Ventriculo-arterial coupling: concepts, assumptions, and applications. Ann Biomed Eng. (1992) 20(1):41–62. 10.1007/BF02368505 1562104

[51] Starling MR. Left ventricular-arterial coupling relations in the normal human heart. Am Heart J. (1993) 125(6):1659–66. 10.1016/0002-8703(93)90756-Y 8498308

[52] Narayan HK French B Khan AM Plappert T Hyman D Bajulaiye A Noninvasive measures of ventricular-arterial coupling and circumferential strain predict cancer therapeutics-related cardiac dysfunction. JACC Cardiovasc Imaging. (2016) 9(10):1131–41. 10.1016/j.jcmg.2015.11.024 27085442 PMC5055405

[53] Grakova EV Shilov SN Kopeva KV Berezikova EN Popova AA Neupokoeva MN Anthracycline-induced cardiotoxicity: the role of endothelial dysfunction. Cardiology. (2021) 146(3):315–23. 10.1159/000512771 33596565

[54] Buckwalter JB Clifford PS. The paradox of sympathetic vasoconstriction in exercising skeletal muscle. Exerc Sport Sci Rev. (2001) 29(4):159–63. 10.1097/00003677-200110000-00005 11688788

[55] Moon SH Moon JC Heo DH Lim YH Choi JH Kim SY Increased pulse wave velocity and augmentation index after isometric handgrip exercise in patients with coronary artery disease. Clin Hypertens. (2015) 21:5. 10.1186/s40885-015-0016-7 26893918 PMC4750784

[56] Spronck B Heusinkveld MHG Vanmolkot FH Op’t Roodt J Hermeling E Delhaas T Pressure-dependence of arterial stiffness: potential clinical implications. J Hypertens. (2015) 33(2):330–8. 10.1097/HJH.0000000000000407 25380150

[57] Li VWY Liu APY Ho KKH Yau JPW Cheuk DKL Cheung YF. Resting and exercise arterial dysfunction in anthracycline-treated adult survivors of childhood cancers. Cardio-Oncol. (2018) 4:9. 10.1186/s40959-018-0035-0. eCollection 2018 PMC7048035 32154007

[58] Cifra B Chen CK Fan CPS Slorach C Manlhiot C McCrindle BW Dynamic myocardial response to exercise in childhood cancer survivors treated with anthracyclines. J Am Soc Echocardiogr. (2018) 31(8):933–42. 10.1016/j.echo.2018.02.003 29615292

